# A collaborative large language model for drug analysis

**DOI:** 10.1038/s41551-025-01471-z

**Published:** 2025-09-23

**Authors:** Hongjian Zhou, Fenglin Liu, Jinge Wu, Wenjun Zhang, Guowei Huang, Lei Clifton, David Eyre, Haochen Luo, Fengyuan Liu, Kim Branson, Patrick Schwab, Xian Wu, Yefeng Zheng, Anshul Thakur, David A. Clifton

**Affiliations:** 1 Institute of Biomedical Engineering, Department of Engineering Science, University of Oxford, Oxford, UK; 2 Institute of Health Informatics, University College London, London, UK; 3 Applied Digital Health, Nuffield Department of Primary Care Health Sciences, University of Oxford, Oxford, UK; 4 Big Data Institute, Nuffield Department of Population Health, University of Oxford, Oxford, UK; 5 GlaxoSmithKline, London, UK; 6 Tencent Jarvis Lab, Beijing, China; 7 Medical Artificial Intelligence Laboratory, Westlake University, Hangzhou, China; 8 Oxford Suzhou Centre for Advanced Research, Suzhou, China

**Keywords:** Biomedical engineering, Health care

## Abstract

Large language models (LLMs), such as ChatGPT, have substantially helped in understanding human inquiries and generating textual content with human-level fluency. However, directly using LLMs in healthcare applications faces several problems. LLMs are prone to produce hallucinations, or fluent content that appears reasonable and genuine but that is factually incorrect. Ideally, the source of the generated content should be easily traced for clinicians to evaluate. We propose a knowledge-grounded collaborative large language model, DrugGPT, to make accurate, evidence-based and faithful recommendations that can be used for clinical decisions. DrugGPT incorporates diverse clinical-standard knowledge bases and introduces a collaborative mechanism that adaptively analyses inquiries, captures relevant knowledge sources and aligns these inquiries and knowledge sources when dealing with different drugs. We evaluate the proposed DrugGPT on drug recommendation, dosage recommendation, identification of adverse reactions, identification of potential drug–drug interactions and answering general pharmacology questions. DrugGPT outperforms a wide range of existing LLMs and achieves state-of-the-art performance across all metrics with fewer parameters than generic LLMs.

## Abstract (web summary)

DrugGPT outperforms existing LLMs to answer questions related to drug interactions and recommendations.

## Main

Large language models (LLMs), such as ChatGPT (GPT-3.5), GPT-4^1–3^ and PaLM^4^, have attracted extensive attention owing to their impressive capabilities in understanding and generating human language. The adoption of LLMs to assist physicians in clinical decision-making has received growing research interest in the communities of both artificial intelligence and clinical medicine^5–13^.

**Fig. 1:**
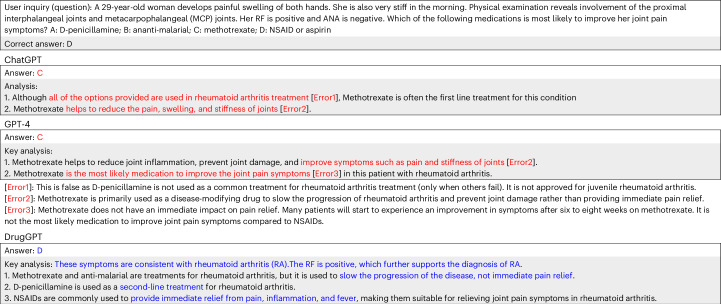
Comparative medical reasoning analysis of LLMs in rheumatoid arthritis treatment. An example drug recommendation provided by DrugGPT and the state-of-the-art LLMs ChatGPT^1^ (GPT-3.5) and GPT-4^2^. The red- and blue-coloured texts denote emphasized wrong and accurate explanations, respectively.

However, applying LLMs in healthcare remains a challenging undertaking owing to the lack of faithfulness and evidence in the generated content^9–12^. The first challenge, faithfulness, means that LLMs tend to produce hallucinations^12,14^, which refer to generated content that looks reasonable but is not based on factual information and knowledge. Given an inquiry that asks for “the medication that is most likely to improve joint pain symptoms (not the first-line treatment for the disease)”, both ChatGPT (that is, GPT-3.5) and GPT-4 not only give a wrong choice ‘C’ but also give wrong explanations without any grounding in accurate knowledge (as shown by the red-coloured text)^15–17^ (Fig. 1). In detail, these hallucinations, for example, “all of the options provided are used in rheumatoid arthritis treatment”, include factually incorrect information that could be explicitly harmful in healthcare decision-making scenarios. The second challenge is the traceability of evidence. In healthcare, it is crucial for LLMs to show the source of generated content (evidence), which can be used to explain why the LLMs give such recommendations. However, existing LLMs often lack the capability to provide clear evidence. Both ChatGPT and GPT-4 give inappropriate evidence (Fig. 1). Such challenges hinder the adoption of LLMs in healthcare settings where transparency, reliability and trustworthiness are critical in achieving trust from clinical users^10,11^, and in regulatory approvals that are essential for deployment in clinical practice.

**Fig. 2:**
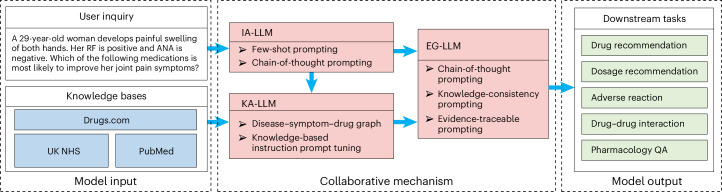
The system architecture of DrugGPT for comprehensive pharmaceutical information processing. A flowchart of the proposed DrugGPT for drug recommendation, dosage recommendation, adverse reaction, drug–drug interaction and pharmacology question answering (QA). The system integrates specialized modules for user inquiry analysis, knowledge acquisition from trusted databases and evidence-based response generation to deliver accurate pharmaceutical information.

In this Article, we propose a knowledge-grounded collaborative LLM that can deal with tasks common in clinical practice (Fig. 2): First, DrugGPT can recommend appropriate drug treatment for patients on the basis of their individual diseases, symptoms, clinical signs and relevant investigation results. It can also help to further individualize the dose of the drug to provide maximum efficacy while minimizing the occurrence of adverse effects and side effects. Third, DrugGPT can take the individual characteristics of the patient and the properties of the drug into consideration to identify patterns of adverse reactions associated with drug use. Fourth, DrugGPT is able to use the knowledge on different drugs to predict the effects of known drug combinations. As a result, physicians can better understand the interactions between different drugs and provide guidance for individualized treatment planning. Finally, DrugGPT can answer pharmacology-related questions accurately by extracting comprehensive knowledge from existing databases, supporting decision-making and medication management.

We first incorporate real drug knowledge from three major existing knowledge bases: Drugs.com (https://www.drugs.com), the UK National Health Service (NHS) (https://www.nhs.uk/) and PubMed (https://pubmed.ncbi.nlm.nih.gov/). The inclusion of state-of-the-art knowledge bases ensures faithfulness in the generated content, by avoiding misleading or erroneous recommendations.

DrugGPT builds on a collaborative mechanism, including three mutually cooperative models. An inquiry analysis LLM (IA-LLM) is responsible for analysing inquiries about diseases, symptoms and drugs; it decides what knowledge is required to answer the current inquiry. For the inquiry analysis module of DrugGPT, motivated by the success of Med-PaLM^5^, we incorporate the chain-of-thought (CoT) prompting^18^ and few-shot prompting^1^ strategies to enable the general-purpose LLM^1,19^ to adapt to the medical domain. We observe that the chosen combination of prompting strategies results in an accurate analysis of inquiries. An additional knowledge acquisition LLM (KA-LLM) extracts potentially relevant information from the knowledge bases, providing evidence for the evidence generation stage^20–22^. We first construct a large medical knowledge graph, that is, the disease–symptom–drug graph (DSDG), which models the relationships between diseases, symptoms and drugs, from the knowledge bases. Additionally, we introduce the knowledge-based instruction prompt tuning method^23,24^ to encourage the LLM to efficiently extract knowledge related to the extracted drugs, symptoms and diseases. Then, an evidence generation LLM (EG-LLM) is responsible for generating the answer to the inquiry, on the basis of the identified evidence extracted by the KA-LLM. We again adopt CoT prompting to improve answer quality^18^. We propose two prompting strategies, that is, knowledge-consistency prompting and evidence-traceable prompting, where the former aims to ensure faithfulness and reduce hallucinations and the latter aims to make the source knowledge (evidence) easy to display and trace. In this way, we observe that the credibility of model-generated content is improved. Through the proposed collaborative mechanism, the three introduced LLMs effectively collaborate with each other to encourage that the generated content is accurate and supported by reliable evidence.

**Fig. 3:**
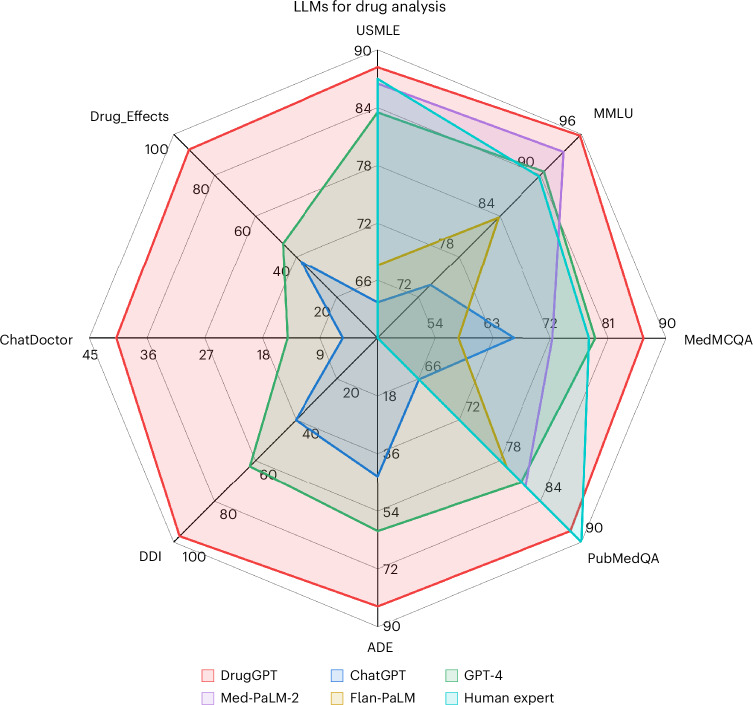
A comparison of the performance of DrugGPT against state-of-the-art language models across eight medical benchmarks. DrugGPT achieves state-of-the-art performance on a broad range of datasets compared with the human expert baseline^25^ and existing strong LLMs, that is, GPT-4^2^, ChatGPT^1^ and Med-PaLM-2^6^. In contrast to existing LLMs, DrugGPT has access to real clinical-standard drug knowledge bases, which considerably increases its ability to answer inquiries accurately. Statistical significance is assessed using two-sided paired *t-*tests, revealing significant differences between DrugGPT and GPT-4 across all benchmarks (USMLE, *P* = 0.031; Medicine, *P* = 0.002; MedMCQA, *P* = 0.012; PubMedQA, *P* = 0.008; ADE, *P* = 5.8 × 10^−8^; DDI, *P* = 3.4 × 10^−6^; ChatDoctor, *P* = 6.5 × 10^−5^; Drug_Effects, *P* = 5.3 × 10^−6^). Source data

We conducted experiments on 11 downstream datasets. As shown in Fig. 3, our proposed DrugGPT outperforms existing strong LLMs, that is, GPT-4^2^, ChatGPT^1^ and Med-PaLM-2^6^, and achieves state-of-the-art results on all tasks. More encouragingly, DrugGPT achieves performance competitive with human experts^25^ on Medical Question Answering (MedQA)-United States Medical License Exams (USMLE)^26^, Massive Multitask Language Understanding (MMLU)-Medicine^27^, Medical Multiple-Choice Question Answering (MedMCQA)^28^ and PubMed Question Answering (PubMedQA)^29^ datasets. These analyses demonstrate the effectiveness of our approach in better assisting physicians than existing LLMs in drug analysis. Finally, we build a user-friendly interface that allows users to input specific requirements to receive personalized answers or recommendations. Additionally, we highlight the underlying knowledge sources employed by DrugGPT.

## Results

In this section, we evaluate the effectiveness of our approach on five downstream tasks, that is, drug recommendation, dosage recommendation, adverse reaction, drug–drug interaction and pharmacology question answering, across 11 datasets. We first describe the evaluation datasets. Then, we illustrate the results of DrugGPT in detail.

To evaluate the performance of DrugGPT, we first adopt eight existing datasets, that is, MedQA-USMLE^26^, MedMCQA^28^, MMLU-Medicine^27^, ChatDoctor^30^, ADE-Corpus-v2^31^, Drug-Effects^32^, DDI-Corpus^33^ and PubMedQA^29^. For current LLMs, in patricular GPT-4 and ChatGPT, the sources of their training data remain unclear. This raises concerns about potential data leakage, where test data may have been used during the training process of the LLMs. To address this potential data leakage issue and evaluate the performance of these LLMs fairly, we further introduced three new datasets^8^: DrugBank-QA, MIMIC-DrugQA and COVID-Moderna. In particular, DrugBank-QA and COVID-Moderna can evaluate the model’s generalizability and transferability to new drugs not covered in the training data^34^. (1) MedQA-USMLE^26^ is a multiple-choice dataset that contains questions and their associated answer candidates. This high-quality dataset is collected from the professional national medical board examination, that is, USMLE. During testing, we randomly select 10,000 questions related to drug recommendation, drug dosage and pharmacology. The LLMs are tasked to select the most appropriate choice. The accuracy can be calculated to report the performance on drug recommendation, dosage recommendation and pharmacology question answering. (2) MedMCQA^28^ is collected from real-world high-quality medical entrance exam questions, covering various healthcare topics and medical subjects. For evaluation, we randomly select 10,000 test samples related to drug recommendation and drug dosage. Each test sample is composed of a question, four choices and a correct answer. Therefore, we ask the LLMs to make the most appropriate choice and calculate the accuracy to report the performance. (3) The MMLU-Medicine^27^ dataset is collected from the MMLU dataset^27^, which is a multitask test consisting of multiple-choice questions from various branches of knowledge, that is, humanities, social sciences, hard sciences and other areas. We selected all samples (that is, 500) from MMLU Professional Medicine and College Medicine to evaluate the performance of our approach on drug recommendation and dosage recommendation. During the evaluation, we asked the LLMs to give the most appropriate choice and calculate the accuracy. (4) The ChatDoctor^30^ dataset is composed of real-world patient–physician conversations in which the conversation describes the disease and symptoms, followed by the final drug recommendations given by the physician. Therefore, we adopt the 500 among 796 test samples and ask the LLMs to list all available drugs for the treatment of patients on the basis of their diseases and symptoms. To evaluate the recommendations made, we calculate the recall, precision and F1 scores. (5) The ADE-Corpus-v2^31^ dataset collected from published medical case reports contains diverse adverse drug reaction data, that is, adverse drug events (ADEs), which provides relations between drugs and adverse effects. We adopt this dataset to evaluate the performance of DrugGPT on the adverse reaction task. Following the conventional ADE evaluations^31^, we randomly selected 5,000 test samples to ask the LLMs to identify the adverse effect mentioned in the input queries. We calculate the accuracy to report the results. (6) Drug-Effects^32^ contains details of various drugs used for diverse medical conditions, for example, acne, cancer and heart disease, and their side effects. This dataset is used for evaluating drug adverse reactions. In detail, we focus on the common effects, that is, the alcohol effect and the pregnancy effect, and randomly extract around 3,000 test samples to ask the LLMs to identify whether the provided drug is proven to have these two effects. The answers given by the LLMs will be in the form of two yes/no choices corresponding to the effects. Therefore, the prediction is correct only if the LLMs correctly identify both effects. We calculate the accuracy to report the results of the LLMs. (7) DDI-Corpus^33^ is a manually annotated dataset containing 5,028 drug–drug interactions (DDIs), including pharmacokinetic and pharmacodynamic interactions. For testing, we build a balanced testing set consisting of 1,000 test samples. In detail, we first randomly selected 500 positive samples with the most common DDIs. Then, we randomly construct 500 negative samples without DDIs, verified by the DDI checker^35^. Therefore, the ratio of positive samples to negative samples is 1:1. Finally, we ask the LLMs to answer ‘yes/no’ for each drug–drug interaction and report the resulting performance. (8) PubMedQA^29^ is a dataset aiming to answer research questions and can verify the reasoning ability of models. We randomly selected 10,000 test samples related to pharmacology to verify the performance on pharmacology question answering. Each test sample consists of a question, a long answer (that is, conclusion) and a short answer (that is, ‘yes’/‘no’). Therefore, we take the question as the inquiry and require the LLMs to give an appropriate short answer (yes/no) and related reasons (conclusions). We calculate the accuracy on the basis of the correctness of the given short answers. (9) For DrugBank-QA, we notice that the GPT-4 and ChatGPT models use training data up to September 2021^36^. Therefore, 213 new drugs released on DrugBank (https://go.drugbank.com/) between October 2023 and April 2024 are used to construct 213 question–answer pairs for evaluation. Each test sample is composed of a question, four choices and a correct answer. As a result, the DrugBank-QA dataset can be used to validate the performance of the LLMs without potential data leakage and to evaluate their performance on new drugs. Accuracy is calculated to report the performance. (10) We further adopt the MIMIC-DrugQA dataset with 1,057 test samples to address the mentioned potential data leakage issue. In detail, we use the data from the MIMIC-IV resource^37^, which integrates de-identified, comprehensive clinical data for patients. MIMIC-IV is a restricted-access resource stored on the Physionet platform^38^ and can only be accessed by a credentialed user who has completed the ‘CITI Data or Specimens Only Research’ training. Therefore, it can be ensured that the MIMIC-DrugQA dataset has not been used during the training process of current LLMs. Each test sample consists of a question related to prescribed medications (for example, ‘What medication should the patient avoid due to her allergy?’), four choices, a correct answer and an associated discharge note. The models are required to choose the correct answer on the basis of the patient’s discharge note. We calculate the accuracy to report the results. (11) COVID-Moderna is adopted to perform the drug–drug interaction task, which involves assessing whether the therapeutic efficacy of the Moderna COVID-19 vaccine (2023 formula) can be decreased when used in combination with other drugs. We use 100 identified drug interactions with the Moderna coronavirus disease 2019 (COVID-19) vaccine and 100 drugs without interaction from ref. ^39^. Therefore, COVID-Moderna is a balanced dataset in which the ratio of positive to negative samples is 1:1. The LLMs are tasked with providing a ‘yes/no’ response for each drug–drug interaction. Similar to the DrugBank-QA dataset, these interactions were identified in 2023 and thus can be used to evaluate the performance of LLMs without potential data leakage and assess their performance on new drugs. We provide the dataset novelty analysis in the Supplementary Information. To facilitate a fair comparison, there is no overlap between the test set and the training data. It is worth noting that, to pre-process each dataset (besides the ChatDoctor dataset), we construct a balanced testing set in which the number of different choices is equal. Therefore, considering the limited computational resources and the allowance of input tokens of GPT-4, the number of test samples selected for each downstream evaluation dataset is determined on the basis of their availability.

### Comparison results

We selected existing state-of-the-art LLMs, that is, Claude-3-Opus^40^, GPT-4^2^, ChatGPT^1^, LLaMA-3-70B^41^, GPT-4-base^42^, Med-PaLM-2^6^, Flan-PaLM^5^, InstructGPT^19^ and Galactica^43^, as the baselines for performance comparison. For Claude-3-Opus, GPT-4 and ChatGPT, to ensure a fair comparison, we adopt the same test data and settings to re-implement their performance on the downstream tasks. For other models, we directly quote the results from their original papers. To ensure that LLMs achieve optimal performance across different downstream tasks, we follow current state-of-the-art works^6,44,45^ to design tailored prompts and instructions for each task (Supplementary Information). We use a strong method, that is, CoT prompting^18^, to improve the performance of baselines. Besides, considering the potential variation in output from ChatGPT and GPT-4, that is, that each run would output a different answer, we perform multiple runs to reduce their randomness and enhance the robustness of the result.

We report the results in Table 1. As we can see, DrugGPT achieves the best performance over all datasets and metrics with fewer parameters. In particular, on the professional national medical board examination USMLE, DrugGPT surpasses the currently popular LLM ChatGPT by 24.5% in accuracy as well as the best LLM GPT-4 by 4.7% in accuracy, with fewer parameters. Besides, on the drug adverse reaction and drug–drug interaction tasks, our method outperforms the existing methods by comfortable margins, achieving absolute improvements of 17.3%, 39.8% and 29.0% in accuracy on the ADE-Corpus-v2^31^, Drug-Effects^32^ and DDI-Corpus^33^ datasets, respectively. The performance of DrugGPT is almost double the performance of ChatGPT and GPT-4. It is worth noting that, on the ChatDoctor dataset, which requires the LLMs to list all potentially appropriate drugs for the treatment of patients, the strong F1 score of DrugGPT demonstrates that DrugGPT can provide more accurate and more comprehensive drug recommendations than previous methods. The promising results prove our arguments and the effectiveness of DrugGPT in providing a better basis for assisting physicians in drug analysis than previous LLMs.

**Table 1:** Performance of different LLMs

Method	No. of parameters	USMLE	MedMCQA	MMLU	ChatDoctor			ADE	Drug_Effects	DDI	PubMedQA
		Accuracy	Accuracy	Accuracy	Precision	Recall	F1	Accuracy	Accuracy	Accuracy	Accuracy
Galactica^43^	120B (0.7×)	44.4	52.9	−	−	−	−	−	−	−	77.6
InstructGPT^19^	175B (1×)	46.0	44.0	35.1	−	−	−	−	−	−	73.2
Flan-PaLM^5^	540B (3.1×)	67.6	57.6	80.1	−	−	−	−	−	−	79.0
Med-PaLM-2^6^	340B (1.9×)	86.5	72.3	89.2	−	−	−	−	−	−	81.8
GPT-4-base^42^	>1T (>5.7×)	86.1	73.7	93.8	−	−	−	−	−	−	80.4
LLaMA-3^41^	70B (0.4×)	_7_9.4 (3.131)	76.0 (3.268)	86.1 (1.769)	14.7 (0.019)	8.0 (0.017)	10.4 (0.020)	56.2 (2.304)	43.3 (0.176)	58.9 (1.679)	78.5 (3.014)
ChatGPT^1^	175B (1×)	63.7 (2.847)	66.3 (2.448)	73.8 (2.181)	6.3 (0.026)	4.7 (0.025)	5.4 (0.027)	43.1 (2.735)	37.5 (0.201)	40.2 (1.191)	66.1 (2.387)
GPT-4^2^	>1T (>5.7×)	83.5 (0.014)	79.0 (0.101)	90.5 (0.025)	19.0 (0.007)	11.2 (0.008)	14.1 (0.009)	60.2 (0.030)	46.5 (0.088)	62.8 (0.062)	81.2 (0.059)
Claude-3^40^	− (−)	86.8 (1.092)	82.3 (0.984)	91.7 (1.120)	24.4 (0.013)	17.2 (0.012)	20.2 (0.013)	66.4 (1.177)	52.0 (0.135)	68.1 (0.398)	84.0 (0.813)
DrugGPT (current work)	175B (1×)	**88.2** (0.044)	**86.5** (0.023)	**95.3** (0.006)	**48.3** (0.207)	**35.3** (0.076)	**40.8** (0.119)	**83.7** (0.024)	**91.8** (0.005)	**97.1** (0.004)	**88.0** (0.005)

‘No. of parameters’ denotes the number of model parameters. We conduct multiple runs for DrugGPT to reduce its randomness. We report the mean (variance) % of performance. All values are reported in percentage (%). Higher is better for all metrics. The multiple in parenthesis in the ‘No. of parameters’ column is calculated by comparison with our method in terms of the number of parameters. Bold values indicate the highest performance in each dataset. Our presented DrugGPT consistently outperforms all previous strong methods over a broad range of datasets.

**Extended Data Fig. 1:**
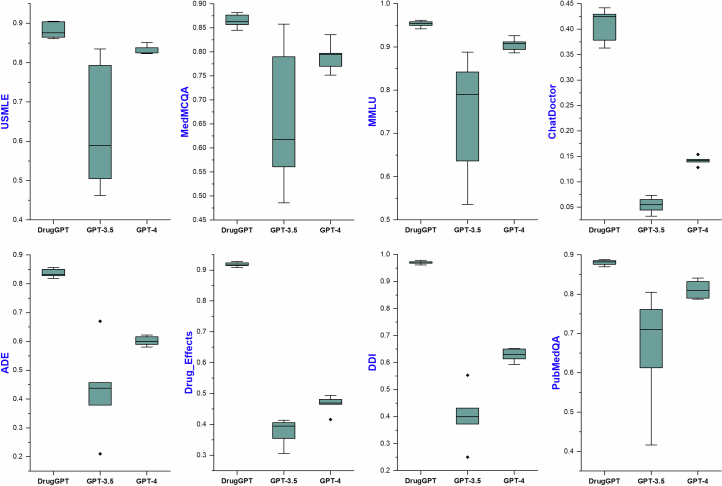
Performance for DrugGPT, ChatGPT, and GPT-4 across 8 benchmarks. We report F1 scores for the ChatDoctor dataset and accuracy for other datasets: USMLE (n=1,000), MedMCQA (n=1,000), MMLU (n=500), ChatDoctor (n=500), ADE (n=1,000), Drug_Effects (n=1,000), DDI (n=1,000), and PubmedQA (n=1,000). Each experiment was independently repeated 5 times with different random seeds (5 independent runs per model-dataset combination). In the boxplots, the central line indicates the median value, while the lower and upper boundaries represent the 25th (Q1) and 75th (Q3) percentiles respectively. The whiskers extend up to 1.5 times the interquartile range (the distance between Q1 and Q3). The unit of analysis was the model performance on each benchmark dataset. Points outside the whiskers represent potential outliers in model performance. Source data

To better understand our approach, we plot the detailed results of DrugGPT, ChatGPT and GPT-4 in Extended Data Fig. 1. As we can see, DrugGPT not only achieves improved performance on all tasks and datasets but also exhibits lower perturbation (that is, deviation) than ChatGPT and GPT-4 across most cases, especially on the DDI datasets used for drug–drug interaction evaluation and the ADE and Drug-Effects datasets used for adverse reaction evaluation. Overall, this result further demonstrates the validity and robustness of DrugGPT in delivering accurate solutions for tasks, resulting in more accurate and robust decision support than existing state-of-the-art LLMs.

### Human evaluation results

For this evaluation, we invited two medical experts to assess the clinical relevance and reliability of LLMs. In detail, we follow previous work^46^ and use the metrics factuality, completeness, safety and preference, which evaluate LLMs’ ability to provide factual, complete, safe and user-preferred information, which is of paramount importance for clinicians^11^. Supplementary Table 1 provides an overview of the metrics.

During the evaluation, we randomly selected 100 samples from the discharge instruction generation task^7,47^, which not only requires LLMs to summarize key diagnostic and medication information but also to generate new clinical text to inform patients about self-management and medication usage. As a result, this task can simultaneously evaluate the LLMs’ abilities to understand and generate clinical texts. Each expert is asked to compare the outputs of our method and that of the baseline models, in terms of the above metrics. The experts are unaware of which LLM generates these outputs.

We select two strong baselines, ChatGPT and GPT-4, to show the human evaluation results, which are reported in Table 2. The results show that our method is better than baselines, offering improved performance. Meanwhile, we can observe that, with fewer model parameters, our DrugGPT outperforms GPT-4 in terms of all metrics, which further shows the effectiveness of our approach in using clinical-standard knowledge bases to provide faithful, accurate and evidence-based recommendations. In particular, our method supports the generation of evidence-traceable content, surpassing ChatGPT and GPT-4 by 76.0 − 13.0 = 63.0 and 68.0 − 18.0 = 50.0 points, respectively, in terms of the preference metric. Overall, our method can enhance the clinical usefulness of LLMs.

**Table 2:** Results of human evaluation, comparing our method with ChatGPT and GPT-4 in terms of factuality, completeness, safety and preference

Metric	ChatGPT^1^ versus DrugGPT			GPT-4^2^ versus DrugGPT		
	ChatGPT wins	Tie	DrugGPT wins	GPT-4 wins	Tie	DrugGPT wins
Factuality	24.0	16.0	60.0	33.0	20.0	47.0
Completeness	15.0	22.0	63.0	21.0	28.0	51.0
Safety	19.0	12.0	69.0	23.0	18.0	59.0
Preference	13.0	11.0	76.0	18.0	14.0	68.0

All values are reported in percentage (%).

### Robustness results

**Fig. 4:**
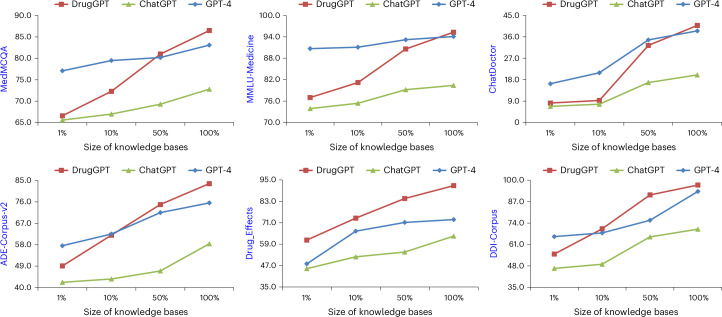
Robustness analysis of DrugGPT. The performance of different methods with respect to various sizes of the knowledge bases used. We report F1 scores for the ChatDoctor dataset and accuracy values for other datasets. In detail, we conduct the evaluations with different sizes of used knowledge bases, from 1% to 100%. DrugGPT consistently outperforms ChatGPT in all cases. With fewer parameters, our method achieves performance similar to GPT-4 when using only a small fraction (that is, 10%) of the available knowledge bases in ADE, Drug-Effects and DDI. Note also that, the more knowledge is used, the better DrugGPT performs compared with baseline models, resulting in its outperforming GPT-4 when using full knowledge bases. Source data

To demonstrate the robustness of DrugGPT to the knowledge base, we evaluate the performance of DrugGPT with respect to the increasing size of the knowledge bases. Figure 4 shows the results of DrugGPT on six datasets. For comparison and a better understanding of the strengths of our proposed method, we further illustrate the performance of ChatGPT and GPT-4. The retriever, top-*k* and chunk size are set to cosine similarity search, 3 and 512, respectively. As we can see, DrugGPT using different sizes of knowledge bases is always better than ChatGPT over all datasets, proving the effectiveness and robustness of our approach to the knowledge base. Using subsets of the available knowledge bases, that is, 10%, DrugGPT is competitive with GPT-4 on the Drug-Effects and DDI datasets. The improved performance under the application scenarios with limited sizes of knowledge bases not only proves our arguments and the robustness of DrugGPT but also shows the potential of our method to be easily applied to other domains (for example, diagnosis and prognosis) by using a small-size specific knowledge base. Moreover, note that, the more knowledge is used, the better DrugGPT performs compared with baseline models. As a result, our DrugGPT can outperform GPT-4 when using full knowledge bases on all downstream tasks. This proves the effectiveness of our method, which can better use knowledge than baseline models.

### Reasoning results

In this evaluation, we performed reasoning analysis for the LLMs to further prove the effectiveness of our method. In implementations, we follow conventional zero-shot learning^5,6^ to require LLMs to perform the task directly on the basis of what it has learned without any references. Zero-shot performance can help quantify the knowledge reasoning ability of the models, such as the ability to directly handle and reason about drugs using the learned knowledge. The zero-shot results are reported in Table 3, showing that DrugGPT achieves the best results across all tasks and datasets with fewer parameters. More encouragingly, DrugGPT outperforms GPT-4 and ChatGPT by around 40% in terms of accuracy on some datasets, for example, DDI, Drug-Effects and ADE datasets. Such promising results encourage users to use DrugGPT for drug analysis more freely and easily.

**Table 3:** Reasoning analysis: evaluations in the zero-shot learning setting to examine whether the LLMs could reason about drugs using learned knowledge

Method	No. of parameters	USMLE	MedMCQA	MMLU	ChatDoctor			ADE	Drug_Effects	DDI	PubMedQA
		Accuracy	Accuracy	Accuracy	Precision	Recall	F1	Accuracy	Accuracy	Accuracy	Accuracy
Med-PaLM-2^6^	340B (1.9×)	79.7	71.3	−	−	−	−	−	−	−	79.2
ChatGPT^1^	175B (1×)	55.8	63.5	71.4	4.7	5.6	5.1	45.2	39.8	42.8	64.1
GPT-4^2^	>1T (>5.7×)	80.2	76.6	84.4	15.6	10.7	12.7	55.5	47.8	58.5	74.5
DrugGPT (current work)	175B (1×)	**82.7**	**80.2**	**85.6**	**50.2**	**33.7**	**40.3**	**84.2**	**92.7**	**95.1**	**84.5**

Bold values indicate the highest performance for each dataset.

We also note that our method outperforms the state-of-the-art medical LLM, Med-PaLM-2. Although our method is designed for drug inquiries, the results show that it can outperform Med-PaLM-2 on general medical answering, such as USMLE, MedMCQA and PubMedQA, with 1.8 times fewer parameters than Med-PaLM-2. Moreover, we provide a solution to enable AI chatbots to accurately understand medical inquiries and deliver evidence-based and faithful medical outputs. Therefore, our method is not limited to drug inquiries; it has the potential to be applied to other medical fields to improve the downstream performance of AI chatbots.

### Results on unseen drugs

We further constructed a DrugQA dataset to evaluate the model’s generalization on unseen types of drugs, which represents a very strong requirement given that, in clinical practice, most drugs on which a system would be queried would be of types that are not entirely unseen with respect to existing drugs.

In detail, the DrugQA dataset, which contains 339 drugs collected from DrugBank, can be divided into three sub-data-sets according to class, type and mechanism of action (MoA)^48^. Supplementary Table 2 summarizes the statistics of the dataset. To validate the model’s generalization to drugs with low similarity, we perform a cross-class/cross-type/cross-MoA prediction by testing the model on one class/type/MoA while training it on other classes/types/MoAs. Taking ‘organic acids’ as an example, we use the drugs that belong to the target class ‘organic acids’ as the testing set and the remaining drugs that do not belong to the target class ‘organic acids’ as the training set. Therefore, there is no overlap of classes/types/MoAs between the training and testing sets. We denote such results as DrugGPT-G. For comparison, we also report the results of DrugGPT, which is tested on the same classes/types/MoAs from the training set. In Table 4, we calculate the accuracy to report the results.

**Table 4:** Generalization analysis of our method

Method	Class							
	Acids	Lipids	Benzenoids	Oxygen	Phenylpropanoids	Nitrogen	Organoheterocyclic	Overall
GPT-4-0613^2^	85.7	89.4	84.4	83.7	**92.7**	87.2	86.1	87.3
GPT-4-1106^2^	90.5	87.2	86.7	88.4	**92.7**	**94.9**	**97.2**	91.4
GPT-4-0125^2^	93.7	85.1	**91.1**	**90.7**	90.2	89.7	91.7	91.2
DrugGPT-G	88.9 (*↓* 6.3)	89.4 (*↓* 2.1)	84.4 (*↓* 4.5)	86.0 (*↓* 4.7)	92.7 (–)	87.2 (*↓* 7.7)	86.1 (*↓* 11.1)	88.5 (*↓* 5.0)
DrugGPT	**95.2**	**91.5**	88.9	**90.7**	**92.7**	**94.9**	**97.2**	**93.5**

Method	Type			Mechanism of action		
	Small molecule	Biotech	Overall	Inhibitor	Antagonist	Overall
GPT-4-0613^2^	87.7	85.7	87.3	86.8	89.5	87.3
GPT-4-1106^2^	91.7	90.5	91.4	86.8	**94.7**	91.4
GPT-4-0125^2^	90.6	93.7	91.2	81.6	**94.7**	91.2
DrugGPT-G	88.4 (*↓* 4.7)	88.9 (*↓* 6.3)	88.5 (*↓* 5.0)	89.5 (*↓* 2.6)	89.5 (*↓* 5.2)	88.5 (*↓* 5.0)
DrugGPT	**93.1**	**95.2**	**93.5**	**92.1**	**94.7**	**93.5**

DrugGPT-G and DrugGPT indicate our method tested on data that is distinct from or from the same classes/types/MoAs as the training set, respectively. (*↓* number) denotes the decrease performance in DrugGPT-G compared with DrugGPT. Bold values indicate the highest performance for each dataset.

As we can see, even for this unusually strong generalization requirement, our approach outperforms the previous GPT-4 baseline models in most cases. Although we observe that the performance of our DrugGPT-G model decreases, DrugGPT-G achieves competitive performance compared with the strong baseline GPT-4-0613. For example, on the ‘acids’, ‘small molecule’ and ‘inhibitor’ splits, DrugGPT-G achieves 88.9%, 88.4% and 89.5% accuracy, which surpasses GPT-4-0613 by 3.2%, 0.7% and 2.7%, respectively. This shows that DrugGPT can generalize well to drugs with lower similarity to those that it saw during training.

## Discussion

We first examine whether LLM forgets the general knowledge it learned previously when encountering new knowledge. In implementations, we adopt the MedQA-USMLE data for testing. We provide four experimental settings: In setting (a), we evaluate the methods with general knowledge from USMLE; in settings (b), (c) and (d), we evaluate the methods with further learned new knowledge from different sources to analyse the impact of the new knowledge on the models’ performance on previous drugs. We can see that our method with new knowledge can still achieve desirable results on previous knowledge, with a slight decrease in accuracy of ~0.4–1.5%, and outperforms GPT-4 and ChatGPT with fewer model parameters (Extended Data Table 1). This indicates that the impact of catastrophic forgetting on our method is limited.

**Extended Data Fig. 2:**
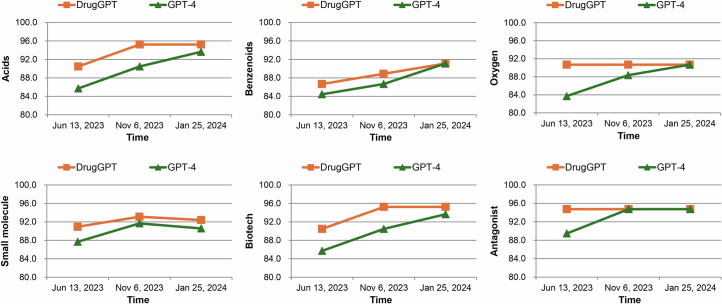
Temporal performance comparison of DrugGPT versus GPT-4 across pharmaceutical knowledge categories. The performances of DrugGPT and GPT-4 are tracked across three time points (June 13, 2023; November 6, 2023; January 25, 2024) for six key pharmaceutical categories: Acids, Benzenoids, Oxygen-containing compounds, Small molecules, Biotech products, and Antagonists. DrugGPT consistently outperforms GPT-4 across all categories and time periods, with both models showing improvement over time. Performance is measured as accuracy percentage on domain-specific pharmaceutical queries. Source data

We now discuss the effect of continual learning in our method. Our work provides a solution to enable LLMs to understand medical inquiries accurately and deliver evidence-based and faithful medical outputs. Our solution is agnostic to basic LLMs; it can be applied to various LLMs to improve their performance on medical tasks. We choose three different variants of GPT-4 models developed at different times^36^: GPT-4-0613 (13 June 2023), GPT-4-1106 (6 November 2023) and GPT-4-0125 (25 January 2024). For comparison, we report the performance of (1) basic LLMs and (2) basic LLMs augmented with our method. We can see that (Extended Data Fig. 2), with the development (that is, continual learning) of GPT-4 models, GPT-4 achieves increasingly better performance in most cases. With our method, the performance of GPT-4 is consistently boosted, and our method outperforms GPT-4 across all variants. The improved results show that our method and continual learning can boost performance in different aspects; combining our method and continual learning achieves an overall improvement.

We further discuss the effectiveness of the proposed collaborative mechanism. All the proposed components, that is, IA-LLM, KA-LLM and EG-LLM, can bring improvements (Extended Data Table 2), proving our arguments and the effectiveness of each component. By comparing settings (a) to (c), we observe that the KA-LLM, which aims to explore related knowledge for LLM, can bring the most improvements, that is, boost the performances from 55.8 to 82.7 on the USMLE dataset, from 63.5 to 80.2 on the MedMCQA dataset and from 71.4 to 85.6 on the MMLU dataset. The achieved improvements further verify the importance of knowledge for drug analysis. More importantly, the designed collaborative mechanism can enable the model to better analyse inquiries, capture relevant broad knowledge and match inquiries and knowledge to generate evidence. As a result, removing any component in the collaborative mechanism would degrade the performance of DrugGPT, underperforming GPT-4. The impaired performance demonstrates that our collaborative mechanism plays an important role in helping DrugGPT outperform GPT-4 with fewer parameters.

**Extended Data Fig. 3:**
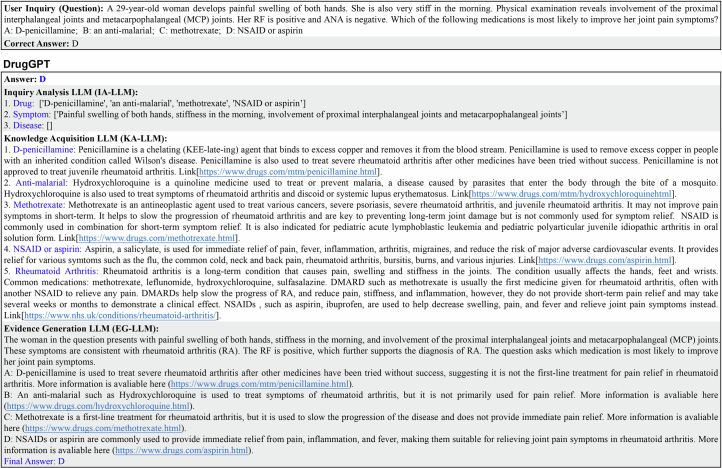
Qualitative study of DrugGPT. The step-by-step reasoning process of DrugGPT demonstrating how the system processes a clinical case. The Inquiry Analysis LLM extracts key drugs and symptoms from the patient case, the Knowledge Acquisition LLM gathers relevant information about each medication option, and the Evidence Generation LLM synthesizes this knowledge to select NSAIDs as the most appropriate treatment. The disease knowledge ‘Rheumatoid Arthritis’ is accurately identified based on the presented symptoms.

We then discuss the reasoning analysis of our model and how it gives the correct answer. The IA-LLM can correctly analyse the input inquiry to extract drugs and symptoms, which can help the model better understand the question (Extended Data Fig. 3). The KA-LLM correctly captures comprehensive knowledge, which can provide accurate evidence to support subsequent model answers and recommendations; for example, the knowledge describing ‘rheumatoid arthritis’ helps the model successfully identify the disease on the basis of the given symptoms of painful swelling of both hands, stiffness in the morning and involvement of specific joints; the knowledge about ‘methotrexate’ provides the information that it is not commonly used for short-term pain relief but rather as a disease-modifying drug to slow down the progression of rheumatoid arthritis and prevent long-term joint damage; the knowledge about ‘NSAIDs’ (non-steroidal anti-inflammatory drugs) highlights that they are commonly used for immediate pain relief, including pain associated with rheumatoid arthritis. Finally, on the basis of the input inquiry (that is, question), which focusses on improving pain symptoms rather than treating the disease itself, the introduced EG-LLM successfully concludes that aspirin (an NSAID) would be the best choice for improving the joint pain symptoms in this patient with rheumatoid arthritis. As a result, on the basis of the knowledge, DrugGPT correctly selects the option and provides the relevant evidence. Overall, DrugGPT can not only provide accurate answers but also generate correct and comprehensive evidence to provide better, trustworthy decision support in comparison with current, state-of-the-art LLMs.

We finally discuss the generalization ability of our method on our three constructed new datasets. As mentioned, since current LLMs have not been specifically trained on these data, we can validate the performance of the LLMs without potential data leakage and demonstrate the effectiveness of DrugGPT in accurately and efficiently dealing with the analysis of new drugs. We can see that our DrugGPT achieves the best results across the three datasets (Extended Data Table 3), demonstrating its improved performance compared with ChatGPT and GPT-4. In particular, on the DrugBank-QA and COVID-Moderna datasets, existing LLMs, that is, ChatGPT and GPT-4, perform poorly (<65% accuracy); fortunately, DrugGPT can achieve decent performance. For example, on the COVID-Moderna dataset, DrugGPT achieves 93.5% accuracy, which surpasses ChatGPT and GPT-4 by 46.5% and 37.0%, respectively. This demonstrates the generalization capability and transferability of DrugGPT, which can be efficiently applied to new drugs to achieve desirable results.

## Methods

In this section, we first briefly formulate the LLM, and then introduce DrugGPT, that is, an ensemble model that incorporates three general-purpose LLMs. Considering that training LLMs is computationally expensive and energy-intensive, we present several fine-tuning/prompting methods to directly adapt existing general-purpose LLMs, for example, GPTs^19^ and LLaMA^49^, to the medical domain. This can enable our method to understand medical inquiries and deliver evidence-based and faithful medical outputs in a data-efficient, parameter-efficient and compute-efficient way, as for Med-PaLM^5^. Supplementary Fig. 1 provides an overview of the prompts and instructions used for each LLM in our model.

### Formulation of LLMs

The great success achieved by LLMs typically relies on the large-scale pre-training strategy. During pre-training, the language model is trained on a massive corpus of open-domain text (that is, wikis and books) in an unsupervised or self-supervised learning manner. The widely used training objectives are masked language modelling (MLM) and autoregressive language modelling (ALM).

#### MLM

Given a sequence of input medical text {*w*_1_, *w*_2_, …, *w*_*N*_} with *N* words, where *w*_*i*_ denotes the *i*th word in the sequence, MLM would randomly mask out a certain percentage (for example, 15%) of the input words, obtaining a sequence of masked words and unmasked words (*w*_m_, *w*_\m_). Then, the LLM is tasked with predicting the randomly masked words *w*_m_ on the basis of the remaining unmasked words *w*_\m_. Therefore, the MLM loss can be defined as follows

1 $${L}^{{{\rm{MLM}}}}=-\mathop{\sum}\limits_{{w}_{{{\rm{m}}}}}\log \left({p}_{\theta }\left({w}_{{{\rm{m}}}}| {w}_{\backslash {{\rm{m}}}}\right)\right),$$

where *p* and *θ* denote the word probability and the model parameters, respectively. The masked tokens are predicted as a classification problem by selecting one word from the vocabulary. As a result, the MLM training objective can encourage the LLM to accurately understand the context and reason for the missing information. The MLM training objective is widely used in bidirectional encoder representations from transformers (BERT)-like LLMs^50^.

#### ALM

is another popular training objective that is widely used in LLMs, for example, ChatGPT^19^ and GPT-3^1^. In implementations, given the context of the previous words *w*_1:*t*−1_ = {*w*_1_, *w*_2_, …, *w*_*t*−1_}, the LLM is trained to predict the next word *w*_*t*_. Therefore, the ALM loss can be defined as

2 $${L}^{{{\rm{ALM}}}}=-\mathop{\sum}\limits_{t=1}^{N}\log \left(\;{p}_{\theta }\left({w}_{t}| {w}_{1:t-1}\right)\right).$$

In this way, ALM can enable the LLM to capture the dependencies and context within the text by modelling the conditional probability distribution of each word in the sequence. The ALM has been widely used in GPT-like LLMs to achieve great success^1^.

#### Inference

During inference, given the input sequence, for example, a user inquiry, *X* = *x*_1:*N*_ = {*x*_1_, *x*_2_, …, *x*_*N*_}, the LLM can generate the subsequent words using the following equation:

3 $$\begin{array}{r}{y}_{1} \sim {p}_{\theta }\left(\;{y}_{1}| {x}_{1:N}\right),\\ {y}_{t} \sim {p}_{\theta }\left(\;{y}_{t}| {x}_{1:N};{y}_{1:t-1}\right),\end{array}$$

where ~ denotes maximum probability sampling from the vocabulary. By iteratively using the above equation, the LLM can output a sequence *Y* = {*y*_1_, *y*_2_, …, *y*_*M*_} for the input sequence, where *M* denotes the length of the output sequence. Finally, the output sequence can be taken as the response to the input user inquiry.

### IA-LLM

We adopt the 175-billion-parameter LLM InstructGPT^19^, as in ChatGPT^1^, as the base module of our approach. In particular, we introduce a unified prompting method to enable the general-purpose LLM to adapt well to the medical domain, resulting in better extraction of relevant medical information from user inquiries and the generation of responses that guide the subsequent LLMs of our ensemble model.

Generally, to adapt deep learning models to a specific domain, transfer learning^51^ and domain adaptation^52^ are widely used. However, they usually rely on a large volume of downstream data for end-to-end fine-tuning^5^. To this end, the few-shot prompting method^1^ is proposed to help LLMs quickly adapt to specific domains with very limited downstream data, for example, few-shot examples, which can be viewed as demonstrations of the target downstream task.

In this way, given the provided few-shot prompts *P* = *p*_1:*L*_ = {*p*_1_, *p*_2_, …, *p*_*L*_} and the current user inquiry *S* = *s*_1:*N*_ = {*s*_1_, *s*_2_, …, *s*_*N*_}, the full input sequence *X* of LLM is the concatenation of user inquiry and prompts

4 $$X=[P;S]=\{{p}_{1},{p}_{2},\ldots ,{p}_{L};{s}_{1},{s}_{2},\ldots ,{s}_{N}\}.$$

Therefore, the total length of the input sequence is *L* + *N*. Then, the output sequence of LLM can be generated by using equation (3).

On the basis of effective few-shot prompting, we further incorporate CoT^18^ prompting to analyse the user inquiries more accurately. For clarity, we show the full prompts of our IA-LLM in Supplementary Table 3.

### KA-LLM

The KA-LLM takes the output of the IA-LLM as input to provide the real knowledge for the following EG-LLM to accurately answer user inquiries. Therefore, the objective of KA-LLM is to extract knowledge from the knowledge bases. This idea is similar to the recently popular retrieval-augmented generation methods^20–22^, which have been demonstrated to be effective in reducing hallucinations and in handling knowledge-intensive tasks^21^. In our model, we first build a large medical knowledge graph, that is, the DSDG, from the major existing medical knowledge bases: Drugs.com, the UK National Health Service and PubMed. Then, we present the knowledge-based instruction prompt tuning to fine-tune the LLM.

#### The DSDG

In this section, to help the LLM better explore medical knowledge from the knowledge bases, as shown in Supplementary Fig. 2, we construct the DSDG to model the relationships between diseases, symptoms and drugs; that is, drugs can treat diseases and diseases are accompanied by symptoms. Therefore, we consider all drugs *V* and all diseases associated with symptoms *U* as nodes; that is, each drug/disease associated with symptoms corresponds to a node in the graph. We denote the DSDG as ${\mathbb{G}}$, including a set of nodes {*U*, *V*} and a set of edges *E*. In implementations, we note that each disease is labelled with a text description that describes the main symptoms of the disease. Similarly, each drug is also labelled with a text description that summarizes the primary diseases that the drug can treat. To obtain the node embeddings, we adopt the MiniLM^53^ with 33 million parameters as the text encoder to extract the text embeddings of their descriptions as the node embeddings. To obtain the edges in the graph, for each disease node ${u}_{i}\in U=\{{u}_{1},{u}_{2},\ldots ,{u}_{{D}_{1}}\}$, we first compute the distance between it and all the nodes of the drugs $V=\{{v}_{1},{v}_{2},\ldots ,{v}_{{D}_{2}}\}$ that can treat the disease. In detail, given a disease node *u*_*i*_ ∈ *U* and a drug node *v*_*j*_ ∈ *V*, their distance *d* is calculated as

5 $$d({u}_{i},{v}_{j})=\frac{\exp \left(\langle {u}_{i},{v}_{j}\rangle /\tau \right)}{{\sum }_{k = 1}^{{D}_{2}}\exp \left(\langle {u}_{i},{v}_{k}\rangle /\tau \right)},$$

where 〈 ⋅, ⋅ 〉 denotes the cosine similarity and *τ* = 0.1 is a temperature hyperparameter. Therefore, the edge weights are calculated by the normalized similarity of different nodes. Similarly, given the drug node *v*_*j*_ ∈ *V*, we can compute the distance between it and all the disease nodes $U=\{{u}_{1},{u}_{2},\ldots ,{u}_{{D}_{1}}\}$ that can be treated by drug *v*_*j*_ as

6 $$d({v}_{j},{u}_{i})=\frac{\exp \left(\langle {v}_{j},{u}_{i}\rangle /\tau \right)}{\mathop{\sum }_{k = 1}^{{D}_{1}}\exp \left(\langle {v}_{j},{u}_{k}\rangle /\tau \right)},$$

Meanwhile, we note that diseases could be treated with many drugs, so to prevent DrugGPT from being overwhelmed by a large number of available drugs and to enable it to prioritize the most relevant drugs for treatment, we choose *K* most relevant drugs with the top-*K* similarity as disease proximity nodes $\hat{V}=\{{\hat{v}}_{1},{\hat{v}}_{2},\ldots ,{\hat{v}}_{K}\}$. As a result, the edge weights can be re-calculated as

7 $$d({u}_{i},{\hat{v}}_{j})=\frac{\exp \left(\langle {u}_{i},{\hat{v}}_{j}\rangle /\tau \right)}{\mathop{\sum }_{k = 1}^{K}\exp \left(\langle {u}_{i},{\hat{v}}_{k}\rangle /\tau \right)}.$$

*K* is a hyperparameter that can be adjusted according to the level of detail required by the downstream tasks. In our work, for simplicity, *K* is set to 5 for all experiments. Similarly, *d*(*v*_*j*_, *u*_*i*_) can be re-calculated as

8 $$d({v}_{j},{\hat{u}}_{i})=\frac{\exp \left(\langle {v}_{j},{\hat{u}}_{i}\rangle /\tau \right)}{\mathop{\sum }_{k = 1}^{K}\exp \left(\langle {v}_{j},{\hat{u}}_{k}\rangle /\tau \right)}.$$

Finally, 13 categories of knowledge, of which 8 are drug knowledge and the remaining 5 are disease knowledge, from Drugs.com, the UK National Health Service and PubMed are linked to each drug in the graph. The eight categories of drug knowledge include ‘drug description and indication’, ‘drug dosage recommendation’, ‘drug adverse effect’, ‘drug toxicity’, ‘drug–food interaction’, ‘drug–drug interaction’, ‘drug pharmacodynamics’ and ‘Pubmed experimental summaries’. The five categories of disease knowledge include ‘common symptoms’, ‘disease causes’, ‘disease diagnosis’, ‘disease treatment’ and ‘disease complications’. For a better understanding of the constructed knowledge graph, we give an illustration in Supplementary Fig. 2.

#### Knowledge-based instruction prompt tuning

We first adopt the widely used graph convolution network^54–56^ as the graph embedding module to acquire the graph embedding *g*_*i*_ for the *i*th node and the global graph embedding *g*_a_, which is obtained by averaging all node embeddings. Then, we calculate the cosine similarity between the text embedding of extracted symptoms and all the disease nodes to extract the disease node with the highest similarity. Finally, we further extract the node embeddings *g*_d_ of the diseases and drugs identified by IA-LLM. In this way, we can adopt the combination of *g*_a_ and *g*_d_ as the initial prefix *g* of KA-LLM to perform knowledge-based instruction prompt tuning, which is proposed to adopt a very small number of data, including pairs of input and output, to adapt the LLM to the downstream task. Therefore, motivated by the success of previous work^5,23,24^, we further construct the task instruction *P* for generation, which is illustrated in Supplementary Table 4. In detail, the task instruction describes the details of how to accurately generate the expected output on the basis of the user inquiry. Finally, we combine the graph embedding *g*, the instruction *P* and the user inquiry *S* together as the input, that is, *X* = {*g*; *P*; *S*}. Since the objective of the LLM is to navigate the knowledge graph and extract the final knowledge, the output is built with a list of knowledge categories, indicating what types of knowledge the KA-LLM should visit, and a list of extracted knowledge from the knowledge graph. We follow previous works^5,23,24^, for example, Med-PaLM, to perform the prompt tuning experiment. We take *X* as the input, and the answer *Y* = *y*_1:*T*_ = {*y*_1_, *y*_2_, …, *y*_*T*_} as the target output, and then adopt the ALM loss to conduct the tuning as

9 $${L}^{{{\rm{ALM}}}}=-\mathop{\sum }_{t=1}^{T}\log \left(\;{p}_{\theta }\left(\;{y}_{t}| X;{y}_{1:t-1}\right)\right).$$

For clarity, in Supplementary Table 5, we show an example of the input and output of KA-LLM used in tuning. The built input and output are used to tune the LLM to accurately extract knowledge to generate faithful and evidence-based outputs.

### EG-LLM

The final component, that is, EG-LLM, is not only responsible for interacting with the user within DrugGPT but also aims to answer user inquiries using the extracted knowledge accurately and efficiently. To this end, we again employ the GPT as the basis module of our EG-LLM. Inspired by the success of Med-PaLM^5^, which has shown that effective prompting on LLM can achieve remarkable performance in the medical field, we introduce three prompting strategies to adapt LLM to the medical domain, obtaining EG-LLM. In detail, we introduce the CoT, knowledge-consistency and evidence-traceable prompting strategies, which are shown in Supplementary Table 6.

#### CoT prompting

In contrast to the traditional single prompt, for example, *‘*Your task is to answer multiple-choice questions*’*, CoT prompting^18^ introduces a complete paragraph or multiple related sentences as input, which can be organized into a chain of prompts (Supplementary Table 6). This can imitate the human thinking process when solving complex problems involving multi-step logical reasoning. As a result, the CoT approach provides a coherent series of intermediate reasoning steps that can guide the model on how to get to the final answer step by step, allowing the model to output a more accurate answer^57^.

#### Knowledge-consistency prompting

Inspired by the success of the self-consistency prompting strategy^58^, we propose the knowledge-consistency prompting strategy to ensure the generation of faithful content and reduce the generation of illusory content, that is, hallucinations. Instead of asking the LLM to choose the best answer, we require the LLM to answer the inquiry with the most ‘truth’, generating the output with the least out-of-context knowledge. Specifically, we encourage EG-LLM to answer user queries using only the knowledge extracted. Therefore, the knowledge-consistency prompting strategy contains a ‘to do’ prompt, that is, ‘Only use the knowledge provided to answer the inquiry’, and a ‘not to do’ prompt, that is, ‘Do not make assumptions not supported by the provided content. Avoid providing personal opinions or interpretations. Summarize and interpret the content as objectively and accurately as possible’. For clarity, we show an example of the used knowledge-consistency prompting in Supplementary Table 6.

#### Evidence-traceable prompting

In this subsection, we further introduce the evidence-traceable prompting strategy to require the LLM to display the source of evidence, including the knowledge used and the source of knowledge, when analysing the input user query. This can enable clinicians to easily inspect the source of the generated content (that is, evidence) and evaluate the providence of the presented information, thus improving the credibility of model-generated content.

To this end, as shown in Supplementary Table 6, we require the LLM to include the knowledge used and the corresponding source of knowledge (for example, a link) as part of the output when appropriate. Otherwise, the model will not be able to generate evidence-traceable content in its answers.

### Experimental settings

Our method is agnostic to the specific choice of basic language models. We adopt the 175-billion-parameter LLM InstructGPT^19^, as in ChatGPT^1^, as the basic module. The knowledge-based instruction prompt tuning is conducted over 20 epochs with a batch size of 32 and a learning rate of 1 × 10^−3^. We used Python (v3.9.21)2.6.0, PyTorch (v2.6.0), NumPy (v2.0.2) and Transformers (v4.51.3) for our study. We adopt the AdamW optimizer^59^ with a weight decay of 0.01 for parameter optimization. In detail, we perform the knowledge-based instruction prompt tuning on LLaMA-7B^49^ with a soft prompt length of 100. Considering that fine-tuning a LLM requires a large amount of data and computational resources, we keep the parameters of the LLM frozen, as in previous works^5,6^. In particular, we collect 1,000 data samples curated and created from various datasets, including PubmedQA, MedMCQA, ADE-Corpus-v2, DDI-Corpus and Drug-Effects. These datasets cover the five downstream tasks of DrugGPT. Each dataset contributed 200 samples, making up 20% of the total data samples used for instruction tuning. The hyper-parameters *τ* and *K* are set to 0.1 and 5. The experiments were conducted on four NVIDIA A100 80-GB graphics processing units (GPUs).

#### Inclusion and ethics

We use 11 public datasets, for which the necessary patient/participant consent has been obtained and the appropriate institutional forms have been officially archived. The protected health information was de-identified in accordance with the regulations set by the Health Insurance Portability and Accountability Act. Protected health information, such as patient names and birth dates, was removed from structured data sources.

### Reporting summary

Further information on research design is available in the Nature Portfolio Reporting Summary linked to this article.

## Supplementary information

Supplementary Information Supplementary Figs. 1–3, Tables 1–8, data and method details, and Discussion.

Reporting Summary

## Source data

Source Data Fig. 3 Source data for DrugGPT and state-of-the-art language models across eight medical benchmarks.

Source Data Fig. 4 Source data for robustness analysis.

Source Data Extended Data Fig. 1 Statistical source data.

Source Data Extended Data Fig. 2 Source data for temporal results.

## Figures and Tables

**Extended Data Table 1:**
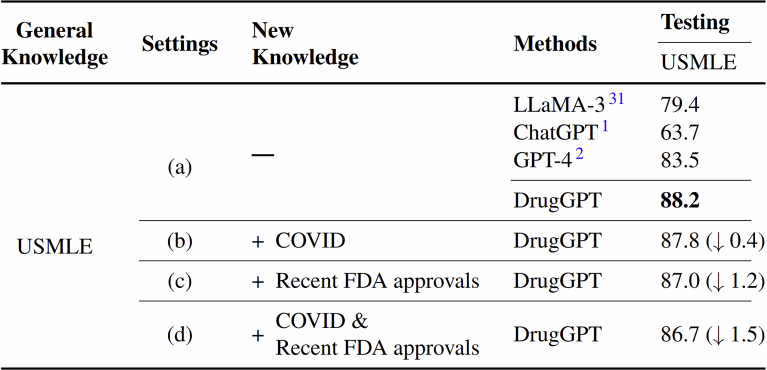
The effect of catastrophic forgetting in our method when encountering new knowledge

We examine whether our method can still provide correct answers for previous drugs. The decreased number denotes the decreased accuracy compared with the best result of our method. Bold values indicate the highest performance.

**Extended Data Table 2:**
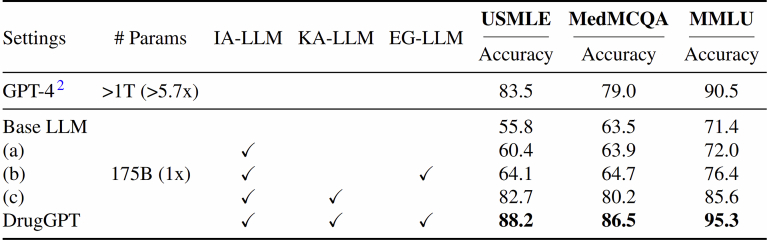
Ablation study of collaborative mechanism in DrugGPT

Our DrugGPT includes three mutually cooperative LLMs, that is, Inquiry Analysis LLM (IA-LLM), Knowledge Acquisition LLM (KA-LLM), and Evidence Generation LLM (EG-LLM). Bold values indicate the highest performance for each dataset.

**Extended Data Table 3:**
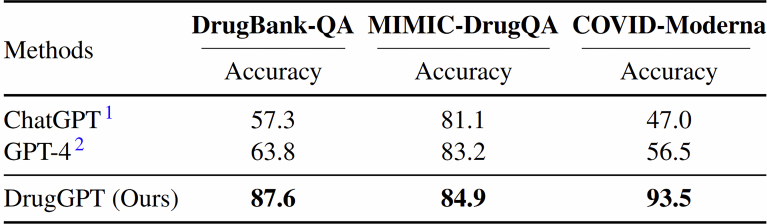
Performances of different LLMs on three new datasets

The three new datasets, that is, DrugBank-QA, MIMIC-DrugQA, and COVID-Moderna, can be ensured to have not been used during the training process of current LLMs. The experiment validates the performance of the LLMs without potential data leakage and examines whether the LLMs can provide decision support for new drugs. We report the average performance. Bold values indicate the highest performance for each dataset.

## Data Availability

We use publicly available data for our experiments. The data supporting the findings of this study are available within the paper. We have also cited all publicly available data on which the findings of the paper rely. The links to download the data used in our work are publicly available via Zenodo at 10.5281/zenodo.15237923 (ref. ^60^). Source data are provided with this paper.

## Code availability

The code for this paper is available via Github at https://github.com/AI-in-Health/DrugGPT (ref. ^60^). Demonstration videos showing DrugGPT performing downstream tasks are available via Loom at https://www.loom.com/share/6528968b9b804db19f5d7c5e1554197a?sid=6b6019e4-d8d5-46b3-bf7d-7c4895ec91bb (DrugGPT recommendation), https://www.loom.com/share/81e82eb651bc4a208097e5f8dc56e28d?sid=a4ca2143-6e6f-48f0-9863-b6e691f40273 (drug and dosage recommendation), https://www.loom.com/share/2b4b91726dfe4a38afe99a56cacd5170?sid=9f0e4480-0b60-48f9-909c-4f1f02c58a82 (adverse reactions), https://www.loom.com/share/e0b43a02862248da937cd10b8c7b0284?sid=3eb53ad4-b248-46a0-8ce4-d1f17389071d (drug–drug interaction), https://www.loom.com/share/5dbac6d2cac9406da717db0572e9d5b6?sid=c8b2827d-533d-42af-ae16-88ecdbb35acc (pharmacology question answering) and https://www.loom.com/share/cc7a476209a444fa851fd4b0bd1ea1fd?sid=18c0f238-1260-4af5-a557-4ca9fb04fef6 (generalization study).
