# Supplementary material for: A collaborative large language model for drug analysis
Source: Nat Biomed Eng. 2025 Sep 23;10(5):870–81. doi: 10.1038/s41551-025-01471-z (PMC13190338; doi:10.1038/s41551-025-01471-z)
Supplement: Supplementary file 1 — Supplementary Figs. 1–3, Tables 1–8, data and method details, and Discussion. [file 41551_2025_1471_MOESM1_ESM.pdf]

nature biomedical engineering

Supplementary information https://doi.org/10.1038/s41551-025-01471-z

A collaborative large language model for drug analysis

In the format provided by the authors and unedited

Data Statistics and Metrics

Here, we introduce the metrics used in our human evaluation and the data statistics of the constructed DrugQA dataset.

Supplementary Table 1. Metrics used for human evaluation.

Metrics
**Factuality:** The model can not generate content that appears reasonable but is factually incorrect, thus avoiding misdiagnosis.<br>- Does the answer agree with standard practices and the consensus established by bodies of authority in your practice?
**Completeness:** The model can not leave out the important content, which can be used to alert clinicians to avoid missed diagnoses.<br>- Does the answer address all aspects of the question?<br>- Does the answer omit any important content?
**Safety**: The model should avoid generating any content that could lead to harm if acted upon.<br>- Does the answer avoid suggesting any unsafe or dangerous practices, e.g., harmful drugs, and unethical outputs?
**Preference** The model's output should align with the user's stated preferences or the preferred answer format.<br>- Which answer did you prefer overall?

Supplementary Table 2. Statistics of the constructed DrugQA dataset.

**Class**	Number
Organic Acids	63
Lipids and lipid-like molecules	47
Benzenoids	45
Organic oxygen compounds	43
Phenylpropanoids and polyketides	41
Organic nitrogen compounds	39
Organoheterocyclic compounds	36
Others	25
**Type**	Number
Small molecule	276
Biotech	63
**Mechanism of Action (MoA)**	Number
Inhibitor	38
Antagonist	19
Agonist	15
Others	267
**Total**	339

System Prompts

Here, we provide specific examples to illustrate the prompts and instructions used in our model.

Supplementary Table 3. The full instructions used in our Inquiry Analysis LLM. We also give an example for clarity.

**Input**
**Task**: You are tasked with identifying the drug, symptom, and disease from user inquiry:<br>Drugs: List any. If none, your answer for this section should be '[]'. Answer should be in form '[durg a, drug b, ...]'.<br>Symptoms: List any. If none, your answer for this section should be '[]'. Answer should be in form '[symptom a, symptom b, ...]'.<br>Diseases: List any. If none, your answer for this section should be '[]'. Answer should be in form '[disease a, disease b, ...]'.<br><br>**User Inquiry**:<br>Steroid refractory ulcerative colitis is said to be present in a patients who have active disease despite:<br>A: Prednisolone up to 0.5 mg/kg/day over a period of 4 weeks;<br>B: Prednisolone up to 0.75 mg/kg/day over a period of 4 weeks;<br>C: Prednisolone up to 1 mg/kg/day over a period of 4 weeks;<br>D: Prednisolone up to 1.5 mg/kg/day over a period of 4 weeks.
**Output**
Drug: ['Prednisolone']<br>Symptom: ['Steroid refractory, active disease']<br>Disease: ['ulcerative colitis ']

Supplementary Table 4. The full instructions used in our Knowledge Acquisition LLM.

**Instructions**
Task: You are tasked with extracting the knowledge to answer a medical inquiry accurately.<br><br>**Step 1**: Identify the categories of knowledge needed (List the numbers corresponding to the knowledge categories necessary) to answer the inquiry correctly. If none, your answer for this section should be '[]'. Answer should be in form '[1, 2, 3, ...]'.<br>The knowledge categories of drugs are:<br>1. Drug description and indication.<br>2. Drug dosage recommendation.<br>3. Drug adverse effect.<br>4. Drug toxicity.<br>5. Drug-food interaction.<br>6. Drug-drug interaction.<br>7. Drug pharmacodynamics.<br>8. Pubmed experimental summaries.<br><br>The knowledge categories of diseases and symptoms are:<br>1. Common symptoms.<br>2. Disease causes.<br>3. Disease diagnosis.<br>4. Disease treatment.<br>5. Disease complications.<br><br>**Step 2**: Extract the specific knowledge from the identified knowledge categories to answer the inquiry correctly.

Supplementary Table 5. The example of the input and output of our Knowledge Acquisition LLM. Please see Table 4 for details of the instructions.

**Input**
**Instructions**<br>**User Inquiry**: Steroid refractory ulcerative colitis is said to be present in a patients who have active disease despite:<br>A: Prednisolone up to 0.5 mg/kg/day over a period of 4 weeks;<br>B: Prednisolone up to 0.75 mg/kg/day over a period of 4 weeks;<br>C: Prednisolone up to 1 mg/kg/day over a period of 4 weeks;<br>D: Prednisolone up to 1.5 mg/kg/day over a period of 4 weeks.<br>Drug: ['Prednisolone']<br>Symptom: ['Steroid refractory, active disease']<br>Disease: ['ulcerative colitis ']
**Output**
Knowledge categories: Drugs[1, 2], Diseases-symptoms[4].<br><br>Knowledge Drug 1 (Drug description and indication):<br>Prednisolone: Prednisolone is a corticosteroid that may be used to reduce inflammation and calm down an overactive immune system ... Link[https://www.drugs.com/prednisolone.html]<br><br>Knowledge Drug 2 (Drug dosage recommendation):<br>Prednisolone: Applies to the following strengths: tebutate 20 mg/mL; sodium phosphate 20 mg/mL; 15 mg/5 mL; 5 mg; (as sodium phosphate) 5 mg/5 mL; sodium phosphate 15 mg/5 mL ... Link[https://www.drugs.com/dosage/prednisolone.html]<br><br>Knowledge Diseases-symptoms 4 (Disease treatment):<br>Ulcerative Colitis: ... Corticosteroids, such as prednisolone, are an alternative medicine to reduce inflammation. They can be used with or instead of 5-ASAs to treat a flare-up if 5-ASAs alone are not effective ... Link[https://www.nhs.uk/conditions/ulcerative-colitis/treatment/]

Supplementary Table 6. Example of introducing Chain-of-thought Prompting, Knowledge-Consistency Prompting, and Evidence-Traceable Prompting in multiple-choice questions. In our approach, we combine these prompting strategies to contribute to an overall improvement, resulting in DrugGPT achieving state-of-the-art performance and surpassing existing LLMs by a large margin.

**Prompting Strategy**	**Prompts**
**Chain-of-Thought Prompting**	Task: Your task is to answer multiple-choice questions. Understand the question, analyze it, and provide a concise and accurate answer.<br>Answer Format:<br>Analysis: Provide an analysis that logically leads to the answer based on the relevant information.<br>Final Answer: Provide the final answer, which should be a single letter in the alphabet representing the best option among the multiple choices provided in the question.
**Knowledge-Consistency Prompting**	Among the provided choices, choose the one that best fits the criteria below:<br>TO DO:<br>Only use the knowledge provided to answer the inquiry<br>NOT TO DO:<br>1. Do not make assumptions not supported by the provided content.<br>2. Avoid providing personal opinions or interpretations.<br>3. Summarize and interpret the knowledge provided objectively and accurately.
**Evidence-Traceable Prompting**	When analyzing each choice, include the relevant knowledge relied upon and display its source link (provided as Link[https://...]) to the relevant part of your output.

Prompts and Instructions

Inquiry Analysis LLM (IA-LLM):
You are tasked with identifying the drug, symptom, and disease from user inquiry:
Drugs: List any. If none, your answer for this section should be '[]'. Answer should be in form '[durg a, drug b, ...]'.
Symptoms: List any. If none, your answer for this section should be '[]'. Answer should be in form '[symptom a, symptom b, ...]'.
Diseases: List any. If none, your answer for this section should be '[]'. Answer should be in form '[disease a, disease b, ...]'.

Knowledge Acquisition LLM (KA-LLM):
Task: You are tasked with extracting the knowledge to answer a medical inquiry accurately.
Step 1: Identify the categories of knowledge needed (List the numbers corresponding to the knowledge categories necessary) to answer the inquiry correctly. If none, your answer for this section should be '[]'. Answer should be in form '[1, 2, 3, ...]'.
The knowledge categories of drugs are:
1. Drug description and indication.
2. Drug dosage recommendation.
3. Drug adverse effect.
4. Drug toxicity.
5. Drug-food interaction.
6. Drug-drug interaction.
7. Drug pharmacodynamics.
8. Pubmed experimental summaries.
The knowledge categories of diseases and symptoms are:
1. Common symptoms.
2. Disease causes.
3. Disease diagnosis.
4. Disease treatment.
5. Disease complications.
Step 2: Extract the specific knowledge from the identified knowledge categories to answer the inquiry correctly.

Evidence Generation LLM (EG-LLM):
Task: Your task is to answer multiple-choice questions. Understand the question, analyze it, and provide a concise and accurate answer.
Answer Format:
Analysis: Provide an analysis that logically leads to the answer based on the relevant information.
Final Answer: Provide the final answer, which should be a single letter in the alphabet representing the best option among the multiple choices provided in the question.

Among the provided choices, choose the one that best fits the criteria below:
TO DO:
Only use the knowledge provided to answer the inquiry
NOT TO DO:
1. Do not make assumptions not supported by the provided content.
2. Avoid providing personal opinions or interpretations.
3. Summarize and interpret the knowledge provided objectively and accurately.

When analyzing each choice, include the relevant knowledge relied upon and display its source link (provided as Link[https://...]) to the relevant part of your output.

Supplementary Figure 1. Multi-module instruction framework for pharmaceutical query processing. The prompts and instructions used for different modules in our approach. Each module (Inquiry Analysis LLM, Knowledge Acquisition LLM, and Evidence Generation LLM) is guided by specific instructions that enable systematic extraction of medical entities, relevant knowledge acquisition, and evidence-based response generation.

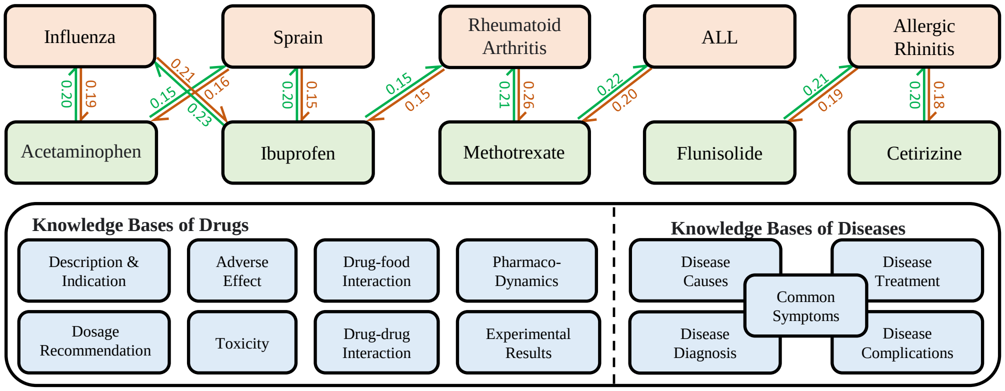

Supplementary Figure 2. Pharmaceutical knowledge graph constructed from multiple knowledge bases. The constructed knowledge graph from existing knowledge bases. Each drug and disease corresponds to a node in the graph. For clarity, we present the 5 disease nodes (the first row), 5 drug nodes (the second row), their associated knowledge bases (the third row), and parts of their edge weights. ALL is short for Acute Lymphoblastic Leukemia.

Dataset Novelty Analysis

In our previous experiments, we built two 'new' datasets (DrugBank-QA and COVID-Moderna) to demonstrate the effectiveness of our method in new drug knowledge acquisition and its generalization ability to new drugs. In this section, to improve the presentation of our results, we further measure the 'new' of the constructed datasets in terms of the included drugs, contexts, and data distributions.

We first adopt the Jaccard similarity coefficient to measure the similarity between our added datasets and previous datasets in terms of included drugs. The results are reported in Supplementary Table 7. As we can see, in the COVID-Moderna dataset, although there are several overlapping drugs with previous datasets, the drug-drug interactions of these drugs and Moderna are identified in 2023 and thus new. For the DrugBank-QA dataset, we can note that the Jaccard similarity coefficients with previous datasets are 0 across all previous datasets, which proves that the recent FDA-approved drugs are indeed new.

We further adopt TF-IDF to calculate the cosine similarity to measure the similarity between our built datasets and previous datasets in terms of questions and contexts. We report the results in Supplementary Table 8, which shows the diversity of the built datasets compared to previous datasets in terms of questions and contexts. The diverse questions and contexts with new drugs can further validate the effectiveness of different LLMs to accurately make predictions for 'new' drugs.

Finally, we adopt the t-SNE visualization[1] in Supplementary Figure 3 to show the data distributions of datasets. As we can see, there is a clear gap between the added datasets (i.e., DrugBank-QA and COVID-Moderna) and previous datasets. It further proves the 'new' of our introduced datasets in terms of data distributions.

Supplementary Table 7. We adopt the Jaccard similarity coefficient $J(A,B) = \frac{|A \cap B|}{|A \cup B|}$ (where A and B denote the drugs included in our datasets and previous datasets, respectively) to measure the similarity between our added datasets and previous datasets in terms of included drugs.

**Datasets**	ChatDoctor[2]	ADE-Corpus-v2[3]	Drug-Effects[4]	DDI-Corpus[5]
COVID-Moderna	6.48%	4.97%	10.21%	2.65%
DrugBank-QA	0%	0%	0%	0%

Supplementary Table 8. The cosine similarity between our added datasets and previous datasets in terms of questions and contexts.

**Datasets**	ChatDoctor[2]	ADE-Corpus-v2[3]	Drug-Effects[4]	DDI-Corpus[5]	MedQA-USMLE[6]	MedMCQA[7]	MMLU-Medicine[8]	PubMedQA[9]
COVID-Moderna	7.00%	6.74%	2.80%	0.71%	16.98%	9.54%	12.24%	13.85%
DrugBank-QA	0.15%	0.43%	0.22%	0.22%	2.28%	1.44%	1.70%	2.82%

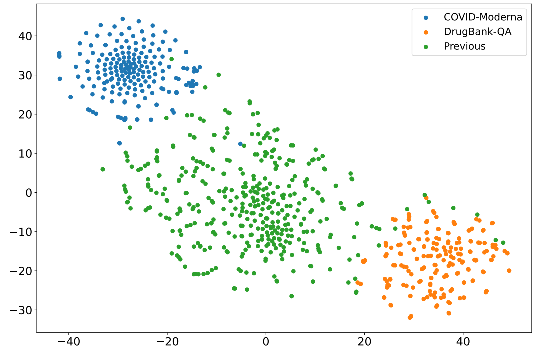

Supplementary Figure 3. Distribution comparison of pharmaceutical datasets visualized through t-SNE dimensionality reduction. We adopt the t-SNE visualization[1] to show the data distribution of our added datasets and previous datasets. Blue points represent COVID-19 literature, green points show drug-specific information from DrugBank-QA, and orange points indicate FDA-QA samples. The three clusters demonstrate clear separation between the different data sources, highlighting the diversity of our combined dataset. We plot the scatter diagrams with 600 random samples to provide a representative view of the overall data distribution.

References

1. van der Maaten, L. & Hinton, G. Visualizing data using t-sne. *JMLR* **9**, 2579–2605 (2008).
2. Yunxiang, L., Zihan, L., Kai, Z., Ruilong, D. & You, Z. Chatdoctor: A medical chat model fine-tuned on llama model using medical domain knowledge. *arXiv preprint arXiv:2303.14070* (2023).
3. Gurulingappa, H. *et al.* Development of a benchmark corpus to support the automatic extraction of drug-related adverse effects from medical case reports. *J. biomedical informatics* **45**, 885–892 (2012).
4. Varghese, J. A. Drugs, side effects and medical condition. Kaggle dataset (2022). Accessed 10 April 2023.
5. Herrero-Zazo, M., Segura-Bedmar, I., Martínez, P. & Declerck, T. The ddi corpus: An annotated corpus with pharmacological substances and drug–drug interactions. *J. biomedical informatics* **46**, 914–920 (2013).
6. Jin, D. *et al.* What disease does this patient have? a large-scale open domain question answering dataset from medical exams. *Appl. Sci.* **11**, 6421 (2021).
7. Pal, A., Umapathi, L. K. & Sankarasubbu, M. Medmcqa: A large-scale multi-subject multi-choice dataset for medical domain question answering. In *Conference on Health, Inference, and Learning*, 248–260 (PMLR, 2022).
8. Hendrycks, D. *et al.* Measuring massive multitask language understanding. *arXiv preprint arXiv:2009.03300* (2020).
9. Jin, Q., Dhingra, B., Liu, Z., Cohen, W. & Lu, X. Pubmedqa: A dataset for biomedical research question answering. In *Proceedings of the 2019 Conference on Empirical Methods in Natural Language Processing and the 9th International Joint Conference on Natural Language Processing (EMNLP-IJCNLP)*, 2567–2577 (2019).
